# Evaluating Vitamin D Deficiency and Toxicity in Indian Children: A Retrospective Study

**DOI:** 10.7759/cureus.82647

**Published:** 2025-04-20

**Authors:** Jyotirmayee Bahinipati, Alpana Mishra, Preetinanda Parida, Ratikanta Tripathy, Nirmal K Mohakud

**Affiliations:** 1 Biochemistry, Kalinga Institute of Medical Sciences, Bhubaneswar, IND; 2 Preventive and Social Medicine, Kalinga Institute of Medical Sciences, Bhubaneswar, IND; 3 Pharmacology, Kalinga Institute of Medical Sciences, Bhubaneswar, IND; 4 Pediatric Medicine, Kalinga Institute of Medical Sciences, Bhubaneswar, IND

**Keywords:** hypercalcemia, hypervitaminosis d, parathyroid hormone, pediatric population, sun exposure, vitamin d deficiency

## Abstract

Background: Vitamin D is essential for bone health, immune function, and overall growth in children. Its deficiency or excess can lead to significant health issues. Despite its importance, there is little systematic data on vitamin D levels in healthy children in Eastern India, with disparities observed across age, gender, and geographical factors. This study investigates the trends in serum vitamin D levels among Indian children, with a specific focus on age- and gender-related variations, and evaluates the implications of current supplementation practices.

Objective: To evaluate vitamin D levels in Eastern Indian children (one month to 18 years) and assess the impact of age, gender, and healthcare settings on vitamin D status.

Methods: A retrospective hospital-based study analyzed vitamin D levels in pediatric patients across different age groups, genders, and healthcare settings (outpatient department (OPD), inpatient department (IPD), and intensive care unit (ICU)). We reviewed data on supplementation practices, clinical manifestations, and underlying conditions, but the retrospective nature of the study limited detailed histories. Data were represented as mean, SD, median, and IQR. An appropriate nonparametric test was applied to find the statistical difference in the data.

Results: Of the total of 1,384 children, the mean age was 11.2 ± 4.9 years. A statistically significant age-related decline was seen (p<0.001) in vitamin D levels. Males had higher vitamin D compared to females (25.75 ± 3.09 vs. 22.85 ± 18.84 ng/ml). Hypovitaminosis D was significantly more prevalent in adolescents aged 11-18 years, with 693 (80.6%) having levels <30 ng/ml (p<0.001). In contrast, hypervitaminosis D (>100 ng/ml) was more common in infants under one year, affecting four (6.5%) (p=0.012). Healthcare settings like OPD, IPD, and ICU did not affect the vitamin D levels significantly.

Conclusion: Vitamin D supplementation in neonates has effectively maintained optimal levels, but careful monitoring is essential to prevent hypervitaminosis. Targeted interventions, especially for adolescents and females, are critical to addressing hypovitaminosis and improving overall vitamin D status in children. A well-defined supplementation protocol, combined with regular screening, can ensure balanced levels in children.

## Introduction

Vitamin D is a fat-soluble vitamin that plays a crucial role in various physiological functions beyond maintaining bone health. Its primary function involves regulating calcium balance and phosphate metabolism, which, along with parathyroid hormone (PTH), supports bone mineralization. The enzyme CYP27B1, responsible for converting 25-hydroxyvitamin D (25(OH)D) into its active form, 1,25-dihydroxyvitamin D (1,25(OH)₂D), is primarily expressed in the kidneys, where it is regulated by FGF-23 and PTH. However, CYP27B1 is also present in several other tissues, including immune cells, keratinocytes, microglia, cardiovascular cells, skin, colon, and breast, where its function is independent of FGF-23 and PTH. This tissue-specific regulation of vitamin D is involved in multiple signaling pathways, producing diverse biological effects. Additionally, an alternative pathway exists, mediated by CYP11A1, which is expressed in the adrenal glands, gonads, placenta, immune cells, and peripheral tissues. The widespread cellular expression of vitamin D plays a key role in regulating various genes associated with tissue differentiation, cellular homeostasis, and activation [1,2]. Vitamin D is essential for immune cell differentiation and proliferation, exerting significant immunomodulatory effects. Its deficiency, along with genetic variations in the vitamin D receptor (VDR), has been linked to autoimmune diseases. Moreover, vitamin D plays a role in immune defense by influencing the monocyte-macrophage lineage. Consequently, vitamin D deficiency increases susceptibility to infections, highlighting its critical role in immune system regulation [3].

Vitamin D also plays a crucial role in regulating metabolic processes, particularly in carbohydrate and lipid metabolism. Insufficient vitamin D levels have been associated with a higher risk of obesity, insulin resistance, and type 2 diabetes mellitus (T2DM) in children and adolescents. Vitamin D is also found to influence cholesterol synthesis and fatty acid oxidation. Studies indicate that children with lower vitamin D levels tend to have a higher body fat percentage, suggesting a possible link to obesity [4]. Research has shown that vitamin D has protective effects on endothelial cells by reducing oxidative stress and inflammation, both of which can lead to vascular dysfunction [4]. Vitamin D is primarily acquired through exposure to sunlight, dietary intake, and supplementation. Several factors, including environment, lifestyle, dietary habits, socio-demographic conditions, and ethnicity, play a significant role in determining an individual’s vitamin D levels [5].

A 2014 systematic review identified vitamin D deficiency and insufficiency (<30 ng/mL) as a major global public health concern, affecting people of all age groups [6]. A 2022 multi-center study revealed that over half of Indian children and adolescents suffer from vitamin D deficiency or insufficiency [7]. This problem persists even in regions with sufficient sunlight and developed countries with long-standing food fortification programs. Globally, it is estimated that around one billion individuals, spanning various ethnicities and age groups, have inadequate vitamin D levels [8]. In India alone, approximately 490 million people are vitamin D deficient, with children and adolescents accounting for 31% of this affected population [9].

With increasing awareness of vitamin D deficiency and its associated health risks, vitamin D supplementation has gained popularity, especially among the general population and pediatric age group [10]. This has eventually led to a parallel scenario of vitamin D toxicity. Vitamin D toxicity can occur across different age groups due to various factors, including variations in drug manufacturing, fortification errors, improper dosing, and excessive intake of high-dose supplements. The most severe cases are often linked to long-term consumption of 50,000 IU or more, particularly in children [11]. Chronic excessive intake may lead to hypercalcemia, causing symptoms such as nausea, vomiting, kidney damage, and other serious health complications. These concerns highlight the need for stringent regulation of vitamin D supplementation and increased public awareness [12,13].

Maintaining optimal vitamin D levels requires regular (six-monthly) monitoring, as both vitamin D deficiency (hypovitaminosis D) and the rising incidence of vitamin D toxicity (hypervitaminosis D; serum 25(OH)D levels >100 ng/mL) pose significant health risks. Ensuring proper regulation through routine assessments, appropriate supplementation, and controlled dietary intake is essential for preventing complications associated with both extremes. This study examines trends in vitamin D levels among Eastern Indian children, focusing on age- and gender-related variations, and highlights the implications of supplementation practices.

## Materials and methods

Study design

The study was a retrospective study conducted by review of laboratory records from November 2022 to October 2024, and data were collected in a case reporting form and entered into Microsoft Office Excel 9 (Microsoft Corporation, Redmond, Washington, United States). The study was conducted at Kalinga Institute of Medical Sciences, Bhubaneswar.

Study population

Children and adolescents aged one month to 18 years with a vitamin D level estimated in the hospital laboratory were included in the study.

Data collection

After obtaining ethical approval from the institutional ethics committee, the laboratory records were reviewed. The relevant information regarding demographic, clinical, and vitamin D levels was recorded. Waiver of consent was applied and approved. Anonymous data was extracted, and confidentiality was maintained. Vitamin D was estimated by competitive immunoassay via the electrochemiluminescence method in Vitros 5600 by Ortho Clinical Diagnostics, Mexico, in the biochemistry section of the central laboratory.

Statistical analysis

The data was entered into MS Excel 2013 and was analyzed using IBM SPSS Statistics for Windows, Version 20 (Released 2011; IBM Corp., Armonk, New York, United States). The number and percentages were reported for nominal data, and the mean ± standard deviation (SD) was reported for quantitative data; for nonparametric data, the median and interquartile range (IQR) were reported. As required, the chi-square, Mann-Whitney U, and Kruskal-Wallis tests were applied for nonparametric data. The p-value of<0.05 was taken to be statistically significant.

Ethical consideration

The study was approved by the Institutional Ethical Committee (IEC) of KIMS (Ref. No: KIMS/KIIT/IEC/544/2021). The study adhered to the ethical principles outlined in the Declaration of Helsinki, ensuring confidentiality, voluntary participation, and the right to withdraw at any stage without consequences.

## Results

A total of 1,384 pediatric patients were included in the study, with a male-to-female ratio of 1.3:1. The mean age of participants was 11.2 ± 4.9 years. Vitamin D levels showed a significant age-related decline, with the highest levels observed in infants under one year (mean ± SD: 37.23 ± 25.81 ng/mL) and the lowest in adolescents aged 11-18 years (mean ± SD: 21.67 ± 17.20 ng/mL; p<0.001). Gender disparities were also evident, with males having significantly higher mean vitamin D levels than females (25.75 ± 3.09 vs. 22.85 ± 18.84 ng/mL; p<0.001) (Table 1). 

**Table 1:** Sociodemographic details and vitamin D distribution among children *Kruskal-Wallis test value (age=113.007); **Mann-Whitney U value (gender=206.703); ***Kruskal-Wallis test value (healthcare category=7.873) OPD: outpatient department; IPD: inpatient department; ICU: intensive care unit

Factors	Distribution	N (%) (N=1384)	Mean ± SD	Median (IQR)	Test statistics value	P-value
Age	<1 yrs	62 (4.5%)	37.23±25.81	31.21 (19.12, 44.47)	113.007*	<0.001^*^
	1-5 yrs	167 (12.1%)	32.37±21.06	26.88 (20.30, 41.80)		
	6-10 yrs	295 (21.3%)	25.11±17.15	21.69 (15.45, 30.30)		
	11-18 yrs	860 (62.1%)	21.67±17.20	17.04 (11.04, 26.64)		
Gender	Male	734 (53.03%)	25.75±3.09	21.50 (13.35, 32.52)	206.703**	<0.001^**^
	Female	650 (46.97%)	22.85±18.84	17.96 (12.17, 26.68)		
Healthcare category	OPD	562 (40.6%)	25.47±18.31		7.873***	0.06^***^
	IPD	732 (52.9%)	23.31±18.32			
	ICU	90 (6.5%)	22.99±2.42			

**Figure 1:**
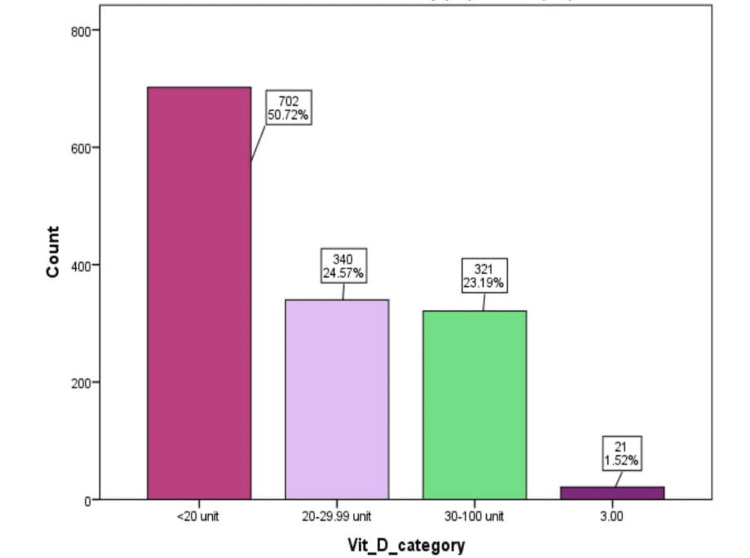
Distribution of vitamin D among children aged one month to 18 years

The majority of the study population, 702 (50.72%), had vitamin D levels below 20 units (ng/ml), indicating widespread deficiency, while only a small fraction, 21 out of 1384 (1.52%), exhibited levels suggestive of toxicity (Figure 1).

**Figure 2:**
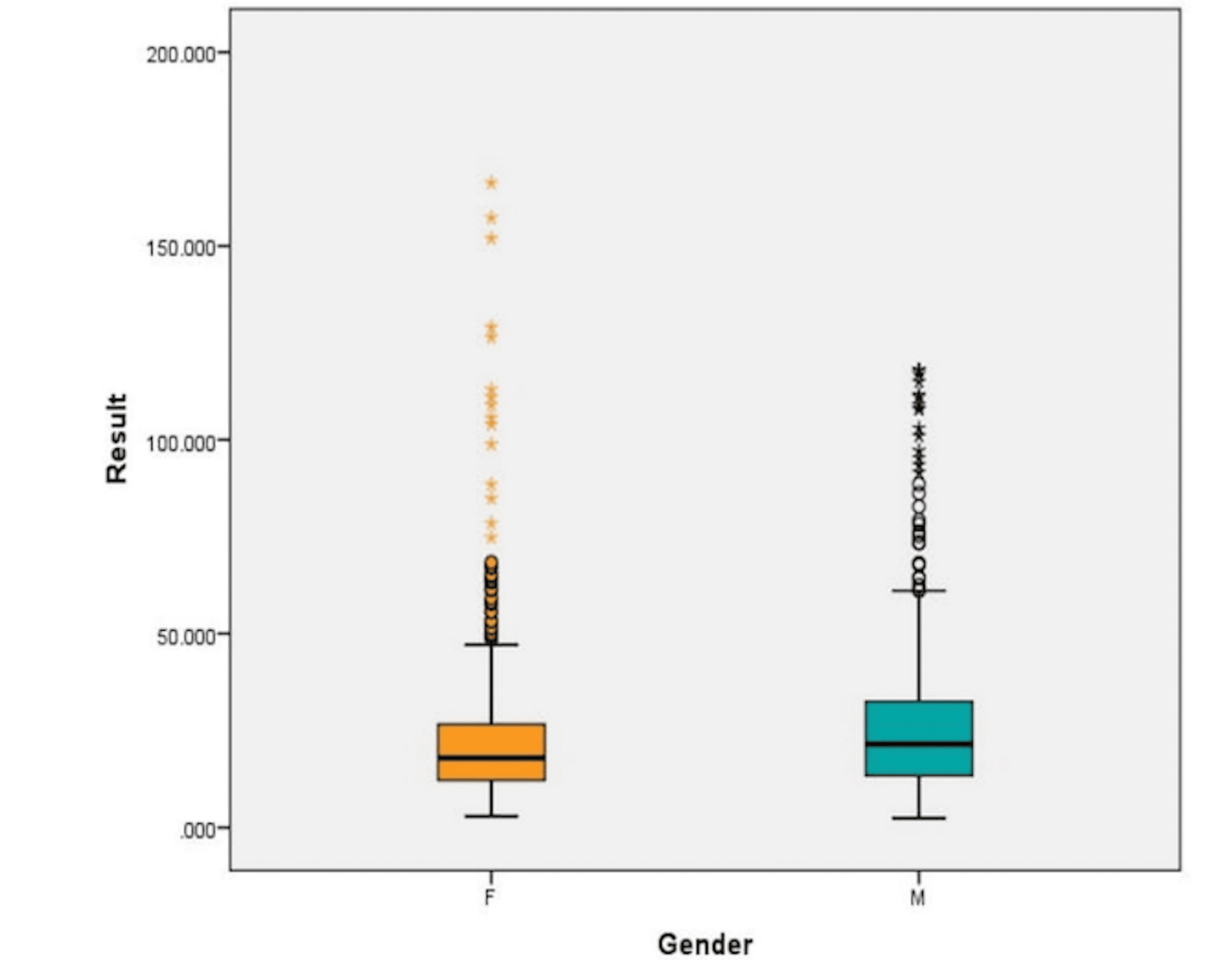
Gender-wise distribution of vitamin D in children, showing females had significantly lower median levels compared to males

The gender-wise distribution shows that females had lower vitamin D levels than males, with more outliers in the female group, with a higher proportion of severe deficiency, highlighting a potential disparity in vitamin D status. The female group also exhibited more outliers, suggesting potential biological (e.g., hormonal differences), behavioral (e.g., sun exposure, clothing habits), or dietary influences (Figure 2).

**Figure 3:**
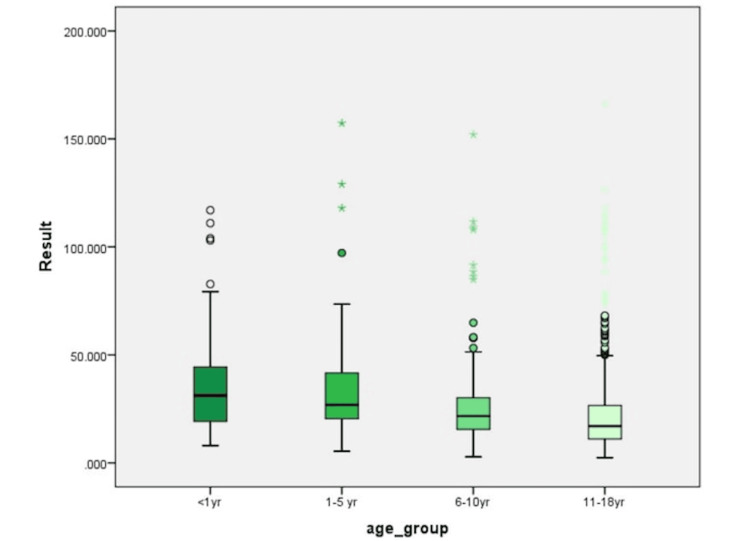
The prevalence of vitamin D deficiency across different pediatric age groups (n=1384) The highest deficiency rates were observed in adolescents (11-18 years) at 80.6%, followed by six to 10 years (73.9%), one to five years (60.5%), and infants <1 year (48.4%).

The age-wise distribution reveals that vitamin D levels are highest in infants (<1 year) and progressively decline with age, reaching the lowest levels in adolescents (11-18 years) (Figure 3).

Hypovitaminosis D in the study population

The prevalence of hypovitaminosis D increases with age, with the highest percentage observed among individuals aged 11 to 18 years (p<0.001). Females exhibited a higher prevalence of hypovitaminosis D at 521 (80.2%) compared to 521 (71.0%) in males (p<0.001). Additionally, hypovitaminosis D was more frequently observed in ICU patients, although this difference was not statistically significant (Table 2). 

**Table 2:** Distribution of hypovitaminosis D in the study population OPD: outpatient department; IPD: inpatient department; ICU: intensive care unit

Factors	Distribution	Vitamin D <30ng/ml N (%)	Vitamin D >=30ng/ml N (%)	Chi-value	P-value
Age	<1 yrs	30 (48.4%)	32 (51.6%)	57.06	<0.001
	1-5 yrs	101 (60.5%)	66 (39.5%)		
	6-10 yrs	218 (73.9%)	77 (26.1%)		
	11-18 yrs	693 (80.6%)	167 (19.4%)		
Gender	Male	521 (71.0%)	213 (29.0%)	15.591	<0.001
	Female	521 (80.2%)	129 (19.8%)		
Health-care category	OPD	412 (73.3%)	152 (26.7%)	4.389	0.11
	IPD	567 (77.5%)	165 (22.5%)		
	ICU	63 (70.0%)	27 (30.0%)		

Hypervitaminosis D in the study population

The prevalence of hypervitaminosis D in the studied population shows that it decreases with age, with the highest levels occurring in infants under one year old. There was no significant difference in prevalence between males and females (p=0.952). Although ICU patients exhibited the highest levels of hypervitaminosis D, this difference was not statistically significant (Table 3).

**Table 3:** Distribution of hypervitaminosis D in the study population OPD: outpatient department; IPD: inpatient department; ICU: intensive care unit

Factors	Distribution	Vitamin D <100 ng/ml N (%)	Vitamin D >100ng/ml N (%)	Chi-value	P-value
Age	<1 yrs	58 (93.5%)	4 (6.5%)	10.964	0.012
	1-5 yrs	164 (98.2%)	3 (1.8%)		
	6-10 yrs	291 (98.6%)	4 (1.4%)		
	11-18 yrs	850 (98.8%)	10 (1.2%)		
Gender	Male	723 (98.5%)	11 (1.5%)	0.004	0.952
	Female	640 (98.5%)	10 (1.5%)		
Health-care category	OPD	555 (98.8%)	7 (1.2%)	2.265	0.322
	IPD	721 (98.5%)	11 (1.5%)		
	ICU	87 (96.7%)	3 (3.3%)		

## Discussion

Our study provides crucial insights into the trends and disparities in vitamin D levels among Indian pediatric patients, highlighting age- and gender-related variations. Notably, vitamin D levels were highest in infants under one year, likely due to routine supplementation, but showed a progressive decline with increasing age, reaching the lowest levels in adolescents. This emphasizes the vulnerability of older children and adolescents to vitamin D deficiency, particularly during periods of rapid skeletal growth. Additionally, we observed a significant gender disparity, with females exhibiting lower vitamin D levels compared to males, underscoring potential sociocultural and lifestyle factors such as reduced outdoor activity and clothing practices that limit sun exposure. Furthermore, while vitamin D toxicity remains rare, our study identified cases of hypervitaminosis in infants, raising concerns regarding potential over-supplementation and the need for vigilant monitoring. These findings reinforce the necessity of age- and gender-specific strategies for vitamin D optimization, including targeted supplementation programs, dietary fortification, and enhanced public awareness.

These findings of our study align with various other studies that indicate older children and adolescents are at increased risk for vitamin D deficiency [7,14]. Levels of vitamin D may be influenced by ethnicity, genetic predisposition, skin type, and sunlight exposure [14]. Besides treatment with certain medications like antiepileptics and glucocorticoids, patients with existing chronic disorders affecting vitamin D absorption (celiac disease) and diseases affecting the liver and kidneys, which prevent vitamin D metabolism, also affect the vitamin D levels [15]. Vitamin D deficiency worsens with age in children due to reduced sunlight exposure (e.g., indoor lifestyles, school demands) and increased physiological demand during rapid adolescent growth, compounded by dietary inadequacies. The lowest levels in adolescents reflect these cumulative stressors, alongside hormonal changes that may impair vitamin D metabolism. 

Infants under one year have a risk for hypovitaminosis, with a greater propensity in premature babies. Breast milk also lacks adequate amounts of vitamin D (10 to 80 IU/L in healthy lactating women) [16]. Deficiency of vitamin D in mothers during the first six months after delivery can even supply a smaller amount to their infants; moreover, infants lack adequate sunlight exposure. Because of the above-mentioned reasons, most international and national guidelines recommend vitamin D prophylaxis in infants during the first year of life to cater to unpredictable, insufficient supply in early infancy [17]. A dose of 400 IU/day is considered safe in preterm infants. Doses of 800 IU/day can achieve the desired biochemical levels faster, but the level of evidence and safety data are not enough to make a separate dosing recommendation for preterm babies. However, the average requirement in older children and adolescents is around 400-600 IU/day, respectively. It is recommended to meet the above requirement through diet and natural sources, like sunlight, in older children. Optimal exposure is direct sunlight (ultraviolet B rays) between 10 AM and 3 PM for 10-30 minutes daily (varies by skin tone: lighter skin ~10 min, darker skin ~30 min), with arms/legs uncovered and no sunscreen. This duration balances synthesis needs with skin cancer risk. Rickets and vitamin D deficiency should be treated with oral cholecalciferol, preferably a daily dosing schedule of 2000 IU below one year of age and 3000 IU in older children for 12 weeks [18]. Weekly high-dose regimens (e.g., 50,000 IU/week for six to eight weeks for adolescents; lower doses adjusted by weight for younger children), followed by daily maintenance (400-1000 IU/day). Monthly dosing (e.g., 100,000-200,000 IU/month) is less common but may be used in specific cases, particularly where adherence to daily/weekly regimens is challenging.

Furthermore, due to insufficient evidence to assess the benefits of supplementation, the WHO has still not recommended supplementation in pregnant women. In our setup, vitamin D is administered to infants as routine supplementation for all breastfed and partially breastfed infants of 400 IU daily to prevent rickets and ensure proper skeletal development. This routine supplementation likely explains the lower prevalence of hypovitaminosis D in this age group.

Under increased need because of skeletal growth and pubertal spurt, the requirement of vitamin D is higher among adolescents [19]. The exact dosage of vitamin D is still not universally acclaimed. Hence, there is an utmost need for age-wise evaluation to take into account various modifiable geographical locations and sun exposure. Routine vitamin D screening in healthy children is currently not recommended globally, which is the reason behind the lack of systematization of vitamin D status in healthy individuals.

Hypervitaminosis, though relatively rare, was primarily detected in infants under one year of age (6.5% n=4) exhibiting serum levels equal to 100 nanograms per ml. This finding raises concern regarding potential overdosage, which may be due to parenteral over-administration, manufacturing differences, and lack of proper monitoring of vitamin D levels [20]. Some case reports highlighted instances where unsupervised vitamin D supplementation led to toxic levels, underscoring the need for careful dosing and routine monitoring of who is receiving supplementation. Hypervitaminosis leads to hypercalcemia, leading to complications such as nephrocalcinosis and renal impairment [21,22]. In addition to age-related trends, gender disparity in vitamin D levels, with females exhibiting lower levels compared to males (p<0.001), has been revealed. This trend has been reported in various Indian populations. Various factors are explained for this disparity: traditional clothing practices, a decrease in engagement of females in outdoor sports, and a reduction in their sun exposure [23]. South Indian-based study done by Harinarayan et al. found nearly 70% of adolescent females had vitamin D deficiency compared to 55% in the male population [24,25]. In a similar study, they found that 90% of school-going females had vitamin D levels below the sufficient range. These findings emphasize that there is an utmost need for targeted intervention, supplementation programs in schools, dietary fortification, and awareness regarding optimum sun exposure [26]. In India, vitamin D-fortified staple foods like chapati flour, maida (refined wheat flour), rice flour, and edible oils are there. Internationally, milk and dairy alternatives (e.g., soy milk), breakfast cereals, margarine, and fruit juices (like orange juice) are commonly fortified with vitamin D, as seen in the U.S. and Canada. Additionally, fortified bread is available in countries like Denmark and Iran.

There are no significant differences in vitamin D status across different healthcare settings: outpatient department (OPD), inpatient department (IPD), and intensive care unit (ICU). However, ICU patients had a higher prevalence of both vitamin D deficiency and toxicity, suggesting the influence of underlying critical illnesses [27,28]. The finding of vitamin D toxicity in pediatric intensive care unit (PICU) patients may be attributed to iatrogenic causes (e.g., high-dose supplementation for deficiency correction in critically ill children) or altered metabolism due to organ dysfunction (e.g., impaired renal clearance, liver failure). Additionally, fluid resuscitation and parenteral nutrition in ICU settings could inadvertently elevate vitamin D levels if not closely monitored.

Monitoring these findings implicates a structured way of approaching vitamin D supplementation. For normal children without risk factors, serum 25(OH)D levels should be checked every six to 12 months, and high-risk groups (e.g., those with malabsorption, obesity, or dark skin) may require more frequent monitoring (every three to six months). Providers thus should emphasize the importance of proper dosing adherence, recommended guidelines, and timely monitoring, particularly in high-risk groups, infants, adolescents, and females.

Limitations

The present study was a retrospective hospital-based database analysis, which inherently limited access to detailed histories regarding comorbidities, chronic disorders, medication use, and lifestyle factors (e.g., sun exposure, diet). To minimize selection bias, we included consecutive eligible cases and applied strict exclusion criteria for incomplete records. However, unmeasured confounders (e.g., socioeconomic status, seasonal variation) could influence vitamin D levels. Recall bias was mitigated by relying on lab-confirmed 25(OH)D values rather than self-reported data. Future prospective studies should systematically capture these variables to validate our findings and explore causal relationships.

## Conclusions

This retrospective study highlights significant age- and gender-related differences in vitamin D levels among children. Vitamin D levels were highest in infants (<1 year) and progressively declined with age, with adolescents (11-18 years) showing the lowest levels. A statistically significant difference was also observed between genders, with males having higher vitamin D levels compared to females. Although no significant difference was noted across healthcare categories (OPD, IPD, ICU), the findings underscore the importance of targeted vitamin D monitoring and intervention strategies, particularly for older children and females who appear to be at greater risk of deficiency. These insights reinforce the need for age- and gender-specific supplementation guidelines and further research into long-term outcomes. Given that routine screening for vitamin D is not currently recommended for healthy children worldwide, there is a clear need for more comprehensive data and standardized guidelines. 
